# Adenoid Cystic Carcinoma of the Lacrimal Gland: High Dose Adjuvant Proton Therapy to Improve Patients Outcomes

**DOI:** 10.3389/fonc.2020.00135

**Published:** 2020-02-18

**Authors:** Paul Lesueur, Etienne Rapeaud, Ludovic De Marzi, Farid Goudjil, Christine Levy, Olivier Galatoire, Pierre Vincent Jacomet, Rémi Dendale, Valentin Calugaru

**Affiliations:** ^1^Radiation Oncology Department, Institut Curie Proton Therapy Center (ICPO), Orsay, France; ^2^Radiation Oncology Department, Institut Curie, Paris, France; ^3^Radiation Oncology Department, Centre François Baclesse, Caen, France; ^4^Normandy University, Université de Caen Basse Normandie, Caen, France; ^5^Ophthalmology Service, Institut Curie, Paris, France; ^6^Ophtalmology Service, Fondation Ophtalmologique Adolphe de Rothschild, Paris, France

**Keywords:** proton-therapy, cystic adenoid carcinoma, lacrymal carcinoma, adjuvant irradiation, radionecrosis

## Abstract

**Introduction:** Lacrymal cystic adenoid carcinoma is a rare disease for which optimal treatment is still debated. In fact, despite aggressive treatment such as eye sparing surgery or orbital exenteration, following by adjuvant radiotherapy, local recurrence and distant metastatic disease are common. This study aims to describe outcomes of eye surgery associated with high dose exclusive adjuvant proton beam irradiation.

**Materials and Methods:** This is a monocentric institutional retrospective study. We retrospectively reviewed records of patients treated in our institution since 2008 with high dose adjuvant proton irradiation for a lacrymal cystic adenoid carcinoma up to a maximum of 75.6Gy(RBE). Other histologies or patients treated with a mix of photon-proton were excluded. A total of 15 patients were finally included.

**Results:** Fifteen patients (80% women, 100% Performance status 0–1) with locally advanced disease (33% T3–T4, 47% R1–R2) were included. After a median follow-up of 67.4 months [13.4–122] the 3 years Overall Survival, local Progression free survival, and progression free survival rates were 78, 70, and 58%, respectively. Six patients exhibited a local recurrence. All patients with conservative surgery maintained their base-line visual acuity and visual field at last follow up. Four patients developed brain radionecrosis.

**Conclusion:** This is the largest series of patients with ACC treated with high dose adjuvant proton therapy. Proton therapy is a safe and efficient treatment and should be considered as an adjuvant irradiation modality to privilege, for patients with lacrimal ACC after conservative or radical eyeball surgery. Dose delivered to temporal lobe should be limited to avoid brain radionecrosis.

## Background

Adenoid cystic carcinoma (ACC) is a malignancy of secretory glands, including salivary glands and more rarely the lacrimal glands. Adenoid cystic carcinoma is the most common malignant epithelial neoplasm of the lacrimal gland (66% of cases) (1). The prognosis of lacrimal variant is classically poorer with a 3.5 folds higher mortality in comparison with the others glands subtypes. Despite aggressive treatment such as eye sparing surgery or orbital exenteration, following by adjuvant radiotherapy, local recurrence and distant metastatic disease are common. Indeed, according to a Surveillance, Epidemiology, and End Results Program (SEER) analysis the median overall survival (OS) was 7.6 years for ACC (2). The substantial morbidity and mortality of lacrymal ACC seems to be due to early perineural invasion and spread along major nerves, as well as along periosteal planes.

Given the scarcity of this tumor, the lack of prospective studies analyzing its treatment, and the limited and mixed results of retrospective studies, the appropriate therapy for local control is under debate.

Similarly as for head and neck ACC, the “gold-standard” treatment consists in radical surgery followed by post-operative radiotherapy (3). The use of adjuvant or concomittant systemic therapy is debated (4). In fact, ACC, whatever the gland involved, is classically considered as a radioresistant tumor, and dose escalation is essential to hope for a curative irradiation. ACC of the lacrimal gland is one of the best clinical presentation to exploit the benefits of proton irradiation, given the highly irregular target volume shape and the need for high dose in the presence of surrounded critical structures.

Previous reports on the effects of proton beam radiation (PBR) on lacrimal gland carcinoma are available but present several limits (5–7): heterogeneous histologic types are included in the same study, patients are treated with a mix of photon/proton irradiation, or ACC subgroup is often a small sample of the cohort. A recent retrospective study reported higher survival and lower recurrence rate, by using modern high dose proton-photon adjuvant irradiation in comparison with historical series, thus, supporting a rationale for high dose proton irradiation (5). In this published study 17 of the 18 included patients received a large dose of PBR (range 52.5 to 60CGE), as well as a smaller dose of photon radiation (range 12 to 23.4 CGE), administered by a linear accelerator (LINAC), for a total radiation dose of 71–76 CGE. The use of a small dose of photontherapy was argue on the fact that photons are more skin-sparing and thus reduces the skin toxicity associated with proton therapy. Photons can also improve the dose conformality.

By reporting the largest retrospective study of patients with lacrymal ACC treated by surgery and high dose post-operative exclusive proton beam irradiation, we aim to show that, in this indication, exclusive high dose proton irradiation is feasible.

## Materials and Methods

### Population and Inclusion Criteria

We screened all patients with lacrymal ACC, treated with proton therapy from 2008 to march 2018, at Institut Curie Proton Therapy Center in Orsay (ICPO). Patients were retrospectively included according to the following inclusion criteria. Patients should have received an adjuvant or exclusive proton irradiation for a localized lacrymal ACC. Metastatic patients at the time of diagnosis were excluded. Diagnosis had to be confirmed by pathologic analysis, with biopsy from the primitive site, or from tumor resection. All patients older than 18 years could be included. Patients with poor performance status (PS), superior to 2, at diagnosis were not included. Tumor staging was based upon clinical information available in the medical records, and is reported according to the pathologic TNM staging of lacrimal gland carcinoma, AJCC 7th edition staging criteria. Patient, tumor, and treatment characteristics were extracted from the medical charts. The treatment of each patient was discussed by a multidisciplinary team.

### Follow Up and Outcomes

Patients were clinically examined every week during the treatment to assess the radiation induced acute toxicity. After irradiation, every 6 months patients were reviewed by their radiation oncologist, and benefited from a full paraclinical examination. At each medical consultation a gadolinium-enhanced MRI and a thoraco-abdominal CT scan were performed. A blood sample looking for a pituitary axis dysfunction was also collected. Every 6 months an ophthalmological visit was scheduled with an evaluation of visual acuity and visual field. The Common Terminology Criteria for Adverse Events, Version 4.0 (CTCAE v4.0) has been used to assess the early and late toxicities. Follow up duration was calculated from the start of proton irradiation to the last clinical visit. The primary outcome was local progression-free survival (LPFS: Patient alive without in-field local recurrence).

### Proton Beam Irradiation

The irradiation was performed for all the patients with 201 MeV protons (cyclotron C230 IBA - 230 MeV). The double scattering technique (DS) was the first technique used for this series of patients, then, patients treated more recently received pencil beam scanning (PBS) irradiation.

**Figure 1:**
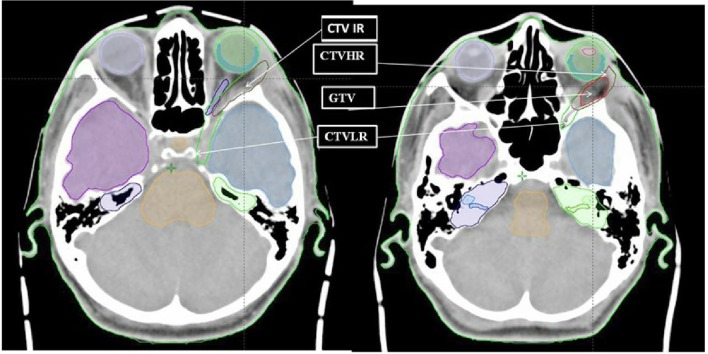
Delineation of HR, IR and LR clinical target volumes.

Patients were positioned supine on the couch and immobilized with a commercial thermoformable mask fixation system. All the patients benefited from a CT scan and a gadolinium-enhanced cerebral MRI, both with 1 mm slice thickness, for registration. The dose limiting structures were the optic nerve, chiasm, ocular globe, retina, brainstem, and temporal lobe. The target volume systematically included the post-surgical residual tumor and tumor bed as well as the sites of possible spread along the optic nerve-path. Three recurrence risk level target volumes were defined: a high, an intermediate and a low risk level. We apply a relative biological effectiveness (RBE) factor of 1.1 to all our prescriptions. In case of only biopsied patients or R2 resection, the high risk CTV 73.8Gy (RBE) (HR-CTV) corresponded to the gross tumor volume with a 3 mm margin, excluding the optic nerve and the ocular globe. In case of R0 or R1 resection, thus the HR-CTV was defined as the operative bed plus 3mm excluding the optic nerve and the ocular globe. The intermediate risk CTV 63Gy(RBE) (IR-CTV) included the ipsilateral half of the orbit, the external wall of orbit and the optic canal while the low risk CTV 54Gy(RBE) (LR-CTV) should encompass the homolateral cavernous sinus (Figure 1). Prophylactic irradiation of cervical lymph node was not proposed. Additional isotropic margins of 1, 2, and 3 mm were added to these volumes to, respectively, define the HR-PTV, the IR-PTV and the LR-PTV. The dose per fraction was 1.8Gy(RBE). For the treatment plans, an anterior oblique ipsilateral field and two superior oblique ipsilateral fields were generally used. For the reduction, the size of the superior oblique fields has been adapted to the different volumes. The dose constraints to the critical organs are summarized in Supplementary Table 1. Coverage of 95% of the target volume by 95% of the prescribed dose was expected.

### Statistical Analysis

Patient's characteristics were described by mean and standard deviation or by median and range for continuous variables and by frequencies for categorical variables. LPFS was defined as the time from the first day of irradiation to the appearance of in-field local failure. Progression-free survival (PFS) and overall survival (OS) were defined as the time from the first day of irradiation treatment to the appearance of recurrence (in field or out-filed) and the death from any cause, respectively. For LPFS, patients not having any evidence of local failure on MRI were censored at last MRI. For PFS and OS, data were censored at the date of death or at the last available clinical encounter. Survival probability was estimated using the Kaplan-Meier method. Univariate analysis using Cox models (for continuous variable) and log-rank tests (for categorical variable) were performed to evaluate the effects of various variables on outcomes. Multivariable assessment was not performed considering the sample size.

### Ethics

This study was approved by French Ethics Committees and the National Commission on informatics and Liberties (MR003 Methodology). An information letter was sent to patients still alive at time of data collection. This study adhered to the Declaration of Helsinki.

## Results

### Patients Characteristics

Between January 2008 and December 2017, 15 patients received a proton irradiation for a lacrymal cystic adenoid carcinoma. All patients were treated at the at Institut Curie Proton Therapy Center in Orsay (ICPO). Twelve patients were women (80%). The median age at diagnosis was 43 years [min-max 23–68]. All patients had a performance status equal to 0 or 1. Two thirds of patients had a localized disease (T2N0M0) while the last third had an advanced local disease (T4 or T3N0M0). Sixty percent of the patients received adjuvant proton therapy after tumorectomy with ocular retention, while for the remaining patients had undergone a proton therapy after an exenteration. Most of patients benefited from a R0 surgery (*n* = 8) while for 7 patients it was reported an incomplete surgery (4 R1 with microscopic involvement margins and 3 R2 with macroscopic residual disease) Patient characteristics are described in Table 1.

**Table 1:** Characteristics of patients included in the study at baseline.

**Characteristics of patients**	
Sex	
Male	3 (20%)
Female	12 (80%)
Age	43 years [23–68]
Performance status	
0–1	15 (100%)
>1	0
TNM stade	
T2N0M0	10 (67%)
T3N0M0	1 (6%)
T4N0M0	4 (27%)
Peri-neural invasion	Yes = 7/ No = 8
Last Surgery before protontherapy	
Upfront exenteration	4 (27%)
Tumorectomy	9 (60%)
Secondary exenteration	2 (13%)
Quality of upfront surgery	
R0	8 (53%)
R1	4 (27%)
R2	3 (20%)
Time from first surgery to protontherapy	91 days [61–1873]
Time from last surgery to protontherapy	96days [61–171]
Irradiation modality	
Double scattering	12 (80%)
Pencil beam scanning	3 (20%)

### Radiotherapy Modalities

Most of patients (*n* = 12) were treated with a double scattering technical (DS), with 3 dose levels as described above and five beams, reduction fields included (median *n* = 5 [4–6]). The high risk area received a median dose of 73.8Gy(RBE) [64–75.6] in 41 fractions of 1.8Gy(RBE). Patients started irradiation 3 months after the last surgery (96days [61–171]). The median proton-therapy duration was 60 days [46–67].

### Progression Free Survival and Overall Survival

**Figure 2:**
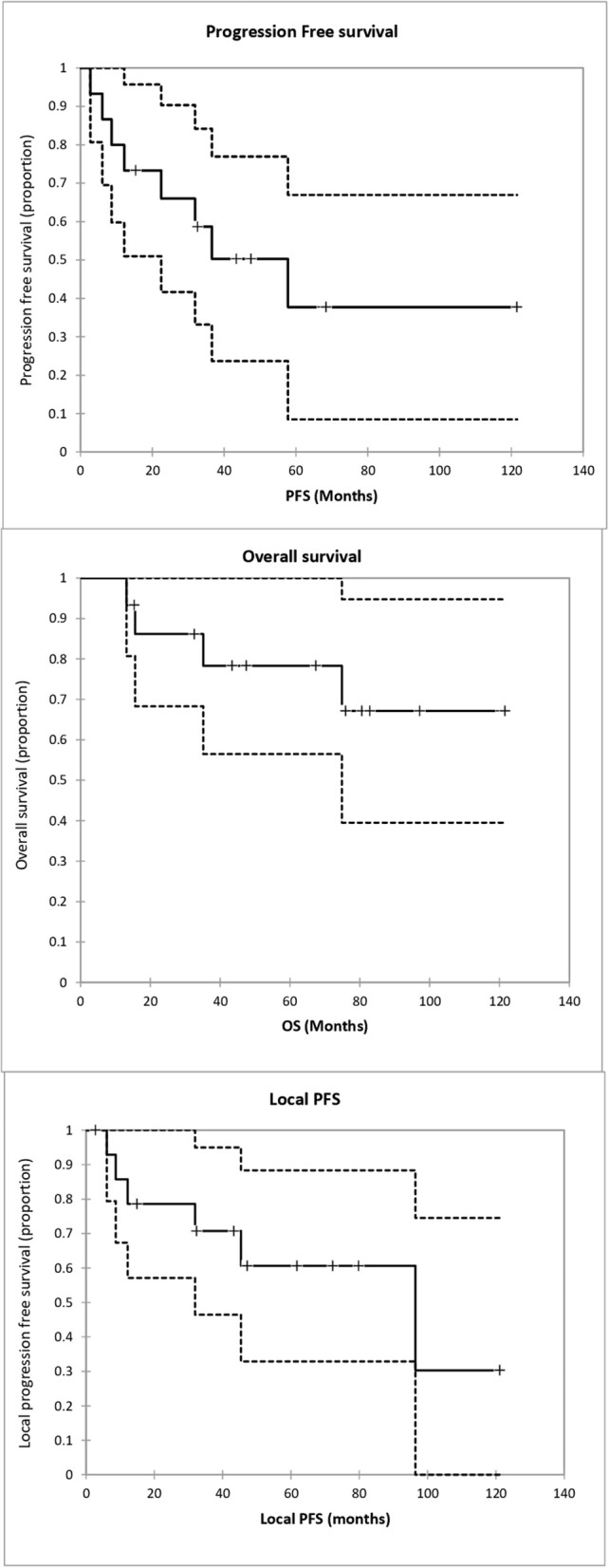
Progression free survival (PFS), Overall Survival (OS) and local progression free survival since irradiation start.

The median follow-up was 67.4 months [13.4–122]. Local progression free survival was 96.1 months [IC95 31.9- NR]. The progression free survival was 57.7 months [IC95 22.5- NR]. Overall survival median was not reached (Figure 2, Table 2).

**Table 2:** Overall and progression free survival rates at 1, 2, 3 and 5 years.

**Overall and progression free survival rates**				
	**1 year**	**2 years**	**3 years**	**5 years**
PFS	80% [IC95% 60–100]	66% [IC95% 41–90]	58% [IC95% 33–84]	38% [IC95% 8–68]
Local PFS	92% [IC95% 87–100]	85% [IC95% 57–100]	70% [IC95% 46–95]	60% [IC95% 32–88]
OS	100%	86% [IC95% 66–100]	78% [IC95% 57–100]	78% [IC95% 57–100]

On univariate analysis, none of the tested factors (sex, age, TNM stage, quality of upfront surgery, volume of high risk CTV, Time From last surgery to irradiation) were predicted of local PFS, PFS, or OS. Only D95% of HR-PTV was predictive of a better overall survival (*p* = 0.05), and showed a trend to a better PFS (*p* = 0.08) (Table 3).

**Table 3:** Prognostic factor (univariate analysis).

	**Local PFS**	**PFS**	**OS**
Sex	0.94	0.96	0.88
Age (>50 vs <50)	0.25	0.38	0.66
TNM (T2 vsT3-T4)	0.95	0.24	0.54
Quality of upfront surgery (R0 vs R1-R2)	0.64	0.83	0.19
Volume of high risk CTV	0.31	0.59	0.23
D95% of high risk PTV	0.43	**0.08**	**0.05**
Time From last surgery to irradiation	0.28	0.20	0.15
Log Rank test for qualitative variable			

### Patterns of Recurrence

Eight patients exhibited a recurrence. Two of them presented a single local relapse, two others patients showed a metastatic relapse, while 4 exhibited both. About local recurrences, 3 intra orbitary relapses were described. All were inside the low risk irradiated volume. Two local recurrences were out of field: inside the infra temporal area for the first one, and along the surgical approach (meningeal fronto-coronal aperture). No patients exhibit recurrences inside the intermediate or high risk irradiated volume. Two patients, treated at first line with adjuvant protontherapy, received a proton reirradiation for their local relapse (after secondary exenteration for the first case, and for an ethmoid relapse in the second one).

### Toxicities

Concerning acute toxicity (during the treatment, or the 3 months following the end of the irradiation), there was no grade III or IV toxicity. All patients presented at least a grade I radiodermitis, and 6 of them a grade II. Others grade I-II symptoms reported, relative to irradiation, were: Nausea(*n* = 4), keratitis (*n* = 1), alopecia (*n* = 2) and peri-orbital oedema (*n* = 1).

Twelve patients developed at least one late radiation-induced toxicity (Table 4). Four patients presented a brain radionecrosis, and for two of them, radionecrosis was symptomatic and needed medication (Grade III according to RTOG scale). One patient developed temporal epilepsy, while the other one presented a brain radionecrosis with a chronic osteitis of the skull bone closest to the involved tumoral site (considered as a grade IV toxicity). This last patient received two proton therapy courses with total dose of 146 Gy (RBE) because of a local recurrence, with no mention of radionecrosis on MRI before the second proton therapy.

**Table 4:** Late secondary effects relative to irradiation.

**Late secondary effects**		
	***n*** **patient**	**Rate (%)**
Brain Radionecrosis	4 (2 grade I, 2 grade III)	27
Xerophtalmia (dry eye)	4 (2 grade II, 2 grade I)	27
Cataracte	1 (grade I)	7
Keratitis	1 (asymptomatic)	7
Hyperprolactinemia	6 (grade I)	40
Pan hypopituitarism	1 (grade I)	7
Osteitis	1 (grade IV)	7

All radionecrosis occurred in patients treated with double scattering irradiation with a prescription dose of 75.6Gy(RBE) delivered to the HR-PTV (*n* = 3) or in patients who received a reirradiation (*n* = 1). For these patients maximal doses delivered to the temporal lobe (at first treatment) were comprised between 73.51Gy(RBE) and 75.7Gy(RBE). Patients with a HR-PTV dose prescription of 73.8Gy(RBE) did not develop any radionecrosis. Dmean and Dmax delivered to the homolateral temporal lobe were 13.06Gy(RBE)[6.75–16.29] and 74.02Gy(RBE)[63.08–75.70].

Post radiation hyperprolactinemia was found for 6 patients, and one patient showed a pan-hypopituitarism which did not require any supplementation. Median Dmax and Dmean delivered to the pituitary were respectively, 53.70 Gy(RBE) [44.07–66.62] and 38.9Gy(RBE) [17.8-53.1]].

At last follow up, all patients with conservative surgery kept a stable bilateral vision compared to pre-proton therapy exams. Four patients presented a chronic grade I-II xeropthalmia. Dmax delivered to the optic chiasm and to homolateral optic nerve were 52.3Gy(RBE)[41.73–55.2] and 60.52Gy(RBE) [58.78–63.78]. Dmean and Dmax delivered to the homolateral ocular globe were 31.74Gy(RBE) [26.3–38.8]] and 66.18Gy(RBE) [55.9–73.7].

## Discussion

To our knowledge we reported here the largest series of patients with ACC treated with high dose adjuvant proton therapy. The overall treatment strategy in our institution consisted in a gross total resection (eye preservative surgery or exenteration) followed by high-dose adjuvant proton-therapy to a median total dose of 73.8 Gy(RBE). Given the retrospective nature of the study, the rare nature of this malignancy and thus the small number of patients in this report, we can only attempt to identify trends; it is not possible to arrive at statistically significant conclusions. It is unfortunately the main limit of our study.

However, our study suggests that high dose adjuvant proton beam irradiation was well-tolerated without high grade acute skin toxicity and produced satisfying rates of local control with limited chronic grade 3 toxicity. Patients benefited from a regular and rigorous audiometric and visual monitoring and None of the 9 patients, still having their eye at irradiation, developed grade III or IV ocular toxicity. Only few patients exhibited manageable grade II keratitis or xerophtalmia. We could however regret the lack of objective in-depth ophthalmologic evaluation such as Optical Coherence Tomography or measure of visual evoked potential. There were probably some patients with infra clinical radiation induced optic ways abnormalities who could not be detected with in routine ophthalmological evaluation.

In the literature, at standard fractionation, a 5 and 10% risk of symptomatic radiation necrosis is predicted to occur at an EQD2 of 72 Gy [range, 60–84] and 90 Gy [range, 84–102] (8, 9). Here, the radionecrosis rate is much higher, since 26% (4/15) of the patients experienced a radionecrosis. For example this rate is closer to that found in patients with locally advanced nasopharyngeal carcinoma treated with radiotherapy alone with a dose escalation (higher than in our study: 81Gy(RBE) vs. 73.8Gy(RBE) (10). This unexpected rates of radionecrosis for the prescribed dose in our study, could be caused by the use of a generic 1.1 RBE value for proton and thus the underestimation of the RBE-weighted dose at the end of proton beams. Indeed, considering our ballistic, high LET could be concentrated in the homolateral temporal lobe, and real biological dose delivered could be underestimated (11). Beddok et al. reported a brain radionecrosis rate of 35% in their series of 17 patients with previously untreated stages III–IVa nasopharyngeal carcinoma (12). These patients were treated with the same protontherapy technical as in our series. These complication is clearly inherent to the protontherapy technical. The use of new tools such as FROG a graphics processing unit (GPU)-based forward calculation tool developed at CNAO (Centro Nazionale di Adroterapia Oncologica) and at HIT (Heidelberg Ion Beam Therapy Center) for fast and accurate calculation of both physical and biological dose could be useful to limit the risk of radionecrosis (13).

The 3 years OS, local PFS and PFS rates were 78, 70, and 58%, respectively. These survival rates are close to these reported by recent retrospectives studies with modern radiotherapy modalities (5, 14, 15). Table 5 reports outcomes and toxicities from 11 recent retrospective studies (5, 14–23). All these series are heterogeneous and small. This makes direct comparisons very difficult. Wolkow (5), reported in 2018, a similar 3 years OS rate of 80% with an adjuvant high dose proton-photon mix irradiation but with higher PFS rate than us (75% for 5years PFS vs. 38% in our study). Nevertheless, our patients had a more advanced disease which could explain the shorter PFS reported in our study in comparison with Wolkow's. Indeed in Wolkow's series, there were one T3 stage and 5 T1a stage whereas in the present study we had 5 T3 or T4 stage without any T1 stage. Ahmad et al. in a series of 53 patients with lacrimal gland adenoid cystic carcinoma, found that tumor size >2.5 cm in greatest dimension correlated with significantly worse disease-free survival compared with smaller tumors (24). This difference between our population and Wolkow's could explain this PFS difference.

**Table 5:** Systematic review of studies dealing with irradiation for lacrymal gland adenoid cystic carcinoma.

**References**	**Patients**	**Follow up**	**Technical**	**Dose (Median)**	**5 years OS**	**5 years PFS**	**Local recurrence rate**	**Systemic recurrence rate**	**Toxicities**
Present study	*n* = 15	5.6 years	Proton	73.8Gy(RBE)	78%	38%	40%	40%	Brain radionecrosis (n = 4)
Sanders et al. (16)	*n* = 8	3.3 years	Photon	72.3Gy	25%	43%	50%	37.5%	Bone exposure (*n* = 2)
Yang et al. (17)	*n* = 24	2.7 years	Photon	Not reported	42%	20%	62.5%	46%	Not reported
Hung et al. (18)	*n* = 11	7.2 years	Photon	60Gy	82%	54.5%	54.5%	36.4%	Radiation neuropathy (*n* = 3), secondary glaucoma (*n* = 3)
Roshan et al. (19)	*n* = 10	1.8 years	Photon	60Gy	100%	71%	10%	10%	Not reported
Wolkow et al. (5)	*n* = 18	12.9 years	Proton + Photon	72Gy(RBE)	85%	75%	22%	17%	Radiation neuropathy (*n* = 5), brain radionecrosis (*n* = 3
Gensheimer et al. (20)	*n* = 11	6.2 years	Neutron	18.4 Neutron Gy	90%	61%	27%	26%	Brain radionecrosis (*n* = 4)
Esmaeli et al. (21)	*n* = 20	2.9 years	Photon	Not reported	56%	Not reported	35%	80%	Not reported
Han et al. (15)	*n* = 10	7.4 years	Photon	60Gy	90%	75%	10%	0%	Radiation retinopathy (*n* = 5)
Noh et al. (14)	*n* = 19	4.8 years	Photon	Median dose 60Gy	83%	65%	26%	11%	Not reported
Wilson et al. (22)	*n* = 7	1.6 years	Photon	Not reported	Not reported	Not reported	0%	29%	Not reported
Natanegara et al. (23)	*n* = 8	Not reported	Photon	Median dose 70Gy	75%	Not reported	0%	50%	Not reported

If at equivalent doses, conventional radiotherapy and protontherapy should lead to the same local control rate, thus why the radiation oncologists should prefer proton-therapy? Late toxicity is expected lower in case of proton beam irradiation, particularly concerning late cognitive impairment. Indeed cognitive deterioration is a largely unrecognized sequela following irradiation of patients with head and neck cancers especially with nasopharyngeal cancer, or sinus carcinoma (25, 26). For example, in Mc Dowell's cross-sectional cohort including 102 long-term nasopharyngeal cancer survivors, impaired MoCA scores (<23) were observed in 32% of patients 7.5 years after IMRT. These patients treated with IMRT showed moderate to high rates of neurocognitive impairment and clinically significant apathy, disinhibition, and executive dysfunction (25). Meyers made the same conclusion for patients with irradiated paranasal sinuses tumors. Over 19 patients, half of the patients had difficulty learning new information, and 80% had accelerated forgetting of the information over time (27). Patients with irradiated lacrimal ACC are exposed to the same risk of cognitive deterioration. In fact frontal and temporal lobes are just behind the target volume. Based on these considerations, proton irradiation is probably the best technical to avoid long term cognitive sequelae, and could be preferred to photon irradiation. Hsiao et al., defined as threshold, a Dmean <36Gy and V60Gy <10% for temporal lobes, to reduce radiation induced cognitive impairment (28). In our population treated with proton therapy, Dmean to the homolateral temporal lobe was 13.06Gy(RBE)[6.75–16.29], far from doses reported in studies with IMRT (10, 26, 28), and may preserve the patients from cognitive disturbance. However, to a avoid cognitive disturbance, practitioners should be very careful with the distribution of high LET in order to reduce brain radionecrosis risk.

The global strategy to cure lacrimal ACC have yet to be determined. If preservative surgery (when possible) and adjuvant radiotherapy are unquestionable, the irradiation modality and the role of intra-arterial neoadjuvant chemotherapy are still discussed (29, 30).

## Conclusion

Given our results, we can conclude that proton therapy is a safe and efficient treatment and should be considered as an adjuvant irradiation modality to privilege, when available, for patients with lacrimal ACC after conservative or radical eyeball surgery, particularly to preserve cognitive structures or contralateral optic pathways.

However, radiation oncologists should pay attention to the volume of brain irradiated with high dose, such as to avoid brain radionecrosis.

## Data Availability Statement

The datasets generated for this study are available on request to the corresponding author.

## Ethics Statement

This study was approved by French Ethics Committees and the National Commission on informatics and Liberties (MR003 Methodology). An information letter was sent to patients still alive at time of data collection. This study adhered to the Declaration of Helsinki.

## Author Contributions

PL wrote the synopsis, the manuscript, computed statistical analyses, and recorded medical data. ER recorded medical data. VC recorded medical data, wrote the synopsis, helped write and improve the manuscript. CL, OG, PJ, and RD were the treating physicians for most of these patients and participated in proofreading. LD and FG were the medical physicists involved in the treatment planification.

### Conflict of Interest

The authors declare that the research was conducted in the absence of any commercial or financial relationships that could be construed as a potential conflict of interest.
