# Supplementary material for: Adenoid Cystic Carcinoma of the Lacrimal Gland: High Dose Adjuvant Proton Therapy to Improve Patients Outcomes
Source: Front Oncol. 2020 Feb 18;10:135. doi: 10.3389/fonc.2020.00135 (PMC7041626; doi:10.3389/fonc.2020.00135)
Supplement: Supplementary file 1 [file Table_1.DOCX]

**Table 1 (Supplementary Material):**

| Dose constraints to critical organs. | |
| --- | --- |
| Organs | Dose constraint EQD2 |
| Ipsilateral Eye-Ball | D0.03 cc ≤ 35 Gy (RBE) |
|  | Dmean ≤ 30 Gy (RBE) |
| Ipsilateral Retina | D0.03 cc ≤ 45 Gy (RBE) |
|  | Dmean ≤ 40 (RBE) |
| Chiasma | D0.03 cc ≤ 54 Gy(RBE) |
| Ipsilateral invaded functional optic nerve | D2% ≤ 60 Gy(RBE) |
| Brainstem | Surface D0.03 cc ≤ 60 Gy (RBE) |
|  | Center D0.03 cc ≤ 54 Gy (RBE) |
|  | Posterieur D0.03 cc ≤ 45 Gy (RBE) |
| Ipsilateral cochlea | D0.03 cc ≤ 50 Gy (RBE) |
| Temporal/Front lobes | V70 Gy (RBE) ≤2 cc |
